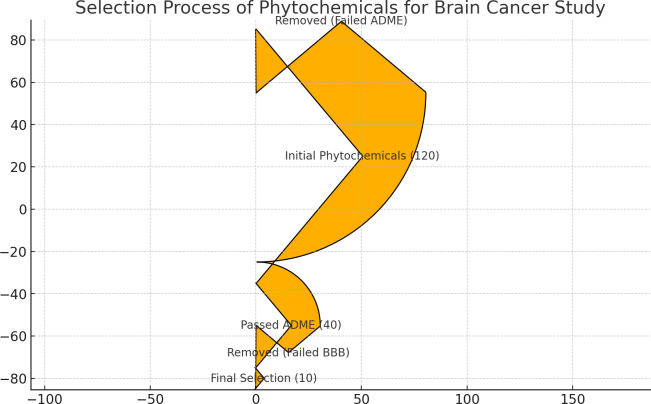# Integrative bioinformatic analysis to identify potential phytochemical candidates for glioblastoma

**DOI:** 10.1016/j.heliyon.2024.e40744

**Published:** 2024-12-05

**Authors:** Hafiza Maria Usmani Rana, Haseeb Nisar, Jignesh Prajapati, Dweipayan Goswami, Ravi Rawat, Volkan Eyupoglu, Samiah Shahid, Anum Javaid, Wardah Nisar

**Affiliations:** a Department of Life-Sciences, University of Management and Technology, Lahore, Pakistan; b Interdisciplinary Research Center for Finance and Digital Economy, KFUPM Business School, King Fahd University of Petroleum and Minerals, Dharan, Saudi Arabia; c Department of Biochemistry & Forensic Science, University School of Sciences, Gujarat University, Ahmedabad, 380009, Gujarat, India; d Department of Microbiology & Biotechnology, University School of Sciences, Gujarat University, Ahmedabad, 380009, Gujarat, India; e School of Health Sciences and Technology, UPES, Dehradun, 248007, India; f Department of Chemistry, Çankırı Karatekin Üniversitesi, Turkey; g Institute of Molecular Biology and Biotechnology, The University of Lahore, Lahore, Pakistan; h School of Biological Sciences, University of the Punjab, Lahore, Pakistan; i Department of Public Health, University of Health Sciences, Lahore, Pakistan

**Keywords:** Glioblastoma, Phytochemicals, Hub genes, Molecular docking, MD simulation

## Abstract

Glioblastoma (GBM) is one of the most malignant forms of cancer with the lowest survival ratio. Our study aims to utilize an integrated bioinformatic analysis to identify hub genes against GBM and explore the active phytochemicals with drug-like properties in treating GBM. The study employed databases of DisGenet, GeneCards, and Gene Expression Omnibus to retrieve GBM-associated genes, revealing 142 overlapping genes. Gene Ontology (GO) and Kyoto Encyclopedia of Genes and Genomes (KEGG) enrichment were used to analyze the role of these genes, which were involved in cancer-associated cell signaling pathways with tyrosine kinase activities and mainly enriched in the Nucleus. Furthermore, the hub genes identification through Cytoscape identified the top 10 ranked genes in a network, which were used as targets to dock against phytochemicals retrieved from the NPACT database having the ability to pass the blood-brain barrier and drug-likeness properties. The molecular docking and dynamics simulation studies predicted the binding of Isochaihulactone and VismioneB to the active site residues of EGFR and SRC genes. In contrast, Resveratrol binds to key residues of PIK3CA. Further, the binding free energy of the docked complex was calculated by performing MM-GBSA analysis, providing a detailed understanding of the underlying molecular interactions. The results offer interactional and structural insights into candidate phytochemicals towards GBM-associated top-ranked proteins. However, validation studies must be done through both in vitro and in vivo disease models to strengthen our computational results.

## 1 Introduction

Glioma, the most prevalent malignant tumor of the central nervous system, represents a significant portion of brain tumor cases, accounting for 51 % of all central nervous system tumors. With an incidence rate of 6.7 per 100,000 globally, the estimated burden in Pakistan is approximately 7097 cases [1]. Gliomas are clinically categorized by the World Health Organization (WHO) into four grades, with grade IV designated as Glioblastoma (GBM), the most aggressive form. GBM is notoriously difficult to treat due to its genetic and epigenetic heterogeneity, which contributes to its resistance to standard therapies and poor prognosis. Additionally, the blood-brain barrier (BBB) poses a significant challenge in delivering therapeutic agents to the brain, further complicating treatment efforts.

Early diagnosis and effective treatment of GBM remain challenging due to the lack of specific diagnostic markers and the disease's rapid progression. Consequently, many patients are diagnosed at advanced stages, reducing the effectiveness of radical surgical interventions and contributing to poor prognoses. The five-year overall survival (OS) rate for GBM patients is alarmingly low, with less than 10 % of patients surviving beyond this period [2]. Despite multimodal treatment strategies, including surgical resection, radiation, and concurrent chemotherapy, the median survival time is only 12–15 months, with two- and five-year survival rates of 25 % and 10 %, respectively [3]. Current diagnostic imaging techniques for GBM lack the specificity needed to distinguish between tumor progression and treatment-related changes, often necessitating further invasive procedures for definitive diagnosis or leading to delays that adversely affect patient outcomes [4]. The limited effectiveness of existing therapies underscores the urgent need for novel, more effective treatments and early diagnostic biomarkers.

Recent advances in gene expression profiling have shown promise in providing more reliable prognostic information compared to traditional histological methods [5]. The identification of hub genes through bioinformatics has emerged as a powerful approach for uncovering potential biomarkers and therapeutic targets in GBM [6]. However, challenges remain in accurately categorizing and predicting GBM outcomes based on molecular biomarkers alone [7]. Advancements in genomics and proteomics have significantly contributed to the discovery of key molecular biomarkers, aiding clinical cancer research in areas such as tumor gene identification, molecular diagnostics, treatment effect assessment, and prognosis prediction [8,9].

Phytochemicals are naturally occurring compounds in plants and represent another option to synthetic drugs due to their possible therapeutic advantages in complementing conventional cancer therapies. Although certain phytochemicals prove to be potent during cancer therapy, this does not protect them from possible side effects. The high structural diversity of phytochemicals introduces a range of novel therapeutic mechanisms, and with due selection and development, they can contribute to treatment regimens that may cause fewer adverse effects compared to synthetic therapies. These compounds have been shown to inhibit cancer cell growth and interfere with cancer progression pathways. Certain phytochemicals may be able to penetrate the blood-brain barrier (BBB) and interact with important signaling pathways linked to the development of glioblastoma (GBM). It is crucial to remember that research is still being done to determine if phytochemicals can successfully target these pathways in GBM. Despite the possibility of therapeutic development, further research is required to ascertain the precise effectiveness, safety profiles, and processes facilitating these substances' trafficking across the blood-brain barrier [10,11]. However, despite the progress, there remains a need for more comprehensive studies that integrate bioinformatics and molecular modeling to identify the most promising phytochemicals for GBM treatment.

In this study, a systematic approach was employed to explore the potential of phytochemicals against glioblastoma (GBM). Initially, GBM-associated genes were identified using GeneCards and DisGenet databases, with further confirmation through Microarray and RNA-Seq analyses. Gene Ontology (GO) and KEGG pathway enrichment analyses were conducted to elucidate the biological roles of these genes in GBM pathology. Key hub proteins were then identified through a protein-protein interaction network, which were subsequently targeted in molecular docking studies. These docking studies, followed by molecular dynamics simulations, assessed the interaction strength and stability of selected phytochemicals from the NPACT database. This comprehensive approach provided detailed insights into the potential of these compounds as effective therapeutic candidates against GBM.

## 2 Materials and methods

### 2.1 Overview of study workflow

This study employed a multi-step approach to identify potential phytochemical inhibitors for glioblastoma (GBM). The process began with the identification of GBM-associated genes, followed by pathway enrichment analysis and the construction of a protein-protein interaction (PPI) network. Key hub proteins were then selected for molecular docking and molecular dynamics (MD) simulations to evaluate the binding affinity and stability of selected phytochemicals, culminating in an analysis of binding energies. The entire workflow is represented in Fig. 1.

**Fig. 1:**
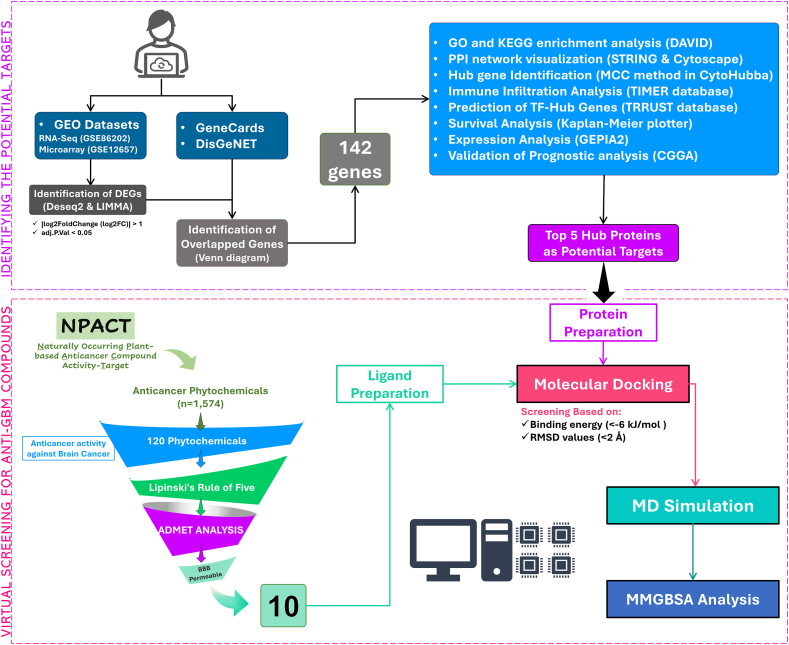
**Workflow.** The figure shows the entire workflow adapted during the study. This includes identifying genes associated with GBM and network analysis followed by GO and Functional enrichment analysis. The phytochemicals were obtained by NPACT database and screened by Molecular Docking and MD Simulation analysis.

### 2.2 Identifying the potential targets

To identify GBM-associated genes, three databases were utilized: DisGeNET, which provides a large collection of genes and variants associated with human diseases, and GeneCards, which compiles gene data from over 90 different sources. Additionally, relevant datasets were obtained from the Gene Expression Omnibus (GEO) using the search term “Glioblastoma.” The inclusion criteria required datasets from Homo sapiens, involving total RNA isolated from GBM tissues and adjacent healthy brain regions, while excluding studies based on cell lines, induced mutations, gene expression therapies, or gene knockdown.

For Microarray analysis, the GSE12657 dataset was selected, and for RNA-Seq analysis, the GSE86202 dataset was used, both meeting the study's inclusion criteria. After normalizing the datasets, differentially expressed genes (DEGs) were identified using the LIMMA package for Microarray data [12] and Deseq2 for RNA-Seq data [13], with thresholds set at |log2FoldChange (log2FC)| > 1 and adj.P.Val <0.05. Duplicate genes were removed, and overlapping genes were visualized using a Venn diagram generated by the ‘Bioinformatics & Evolutionary Genomics’ toolkit (http://bioinformatics.psb.ugent.be/webtools/Venn/).

### 2.3 GO-KEGG pathway enrichment analysis

Gene Ontology (GO) and Kyoto Encyclopedia of Genes and Genomes (KEGG) pathway enrichment analyses were conducted to explore the biological functions and pathways associated with the identified target proteins. The Database for Annotation, Visualization, and Integrated Discovery (DAVID) tools were utilized for this analysis, with a statistical threshold of an adjusted p-value <0.05 applied to select significant GO terms and KEGG pathways [14].

### 2.4 Developing protein-protein interactions (PPI) network

The STRING database was used to examine protein-protein interactions (PPIs) among the selected candidate targets [15]. The analysis was performed with a confidence threshold of 0.700, and a false discovery rate (FDR) stringency of 5 % was applied. The resulting PPI network was then exported to Cytoscape (version 3.9.1) for visualization [16]. The top 10 hub proteins were identified based on Maximal Clique Centrality (MCC) values using the CytoHubba plugin in Cytoscape [17]. These hub proteins were subsequently designated as primary biomarkers for GBM.

### 2.5 Immune infiltration and prediction of transcription factors (TFs)

The TIMER tool (https://cistrome.shinyapps.io/timer) was employed to estimate the abundance of six types of immune cell infiltrates (B cells, CD4^+^ T cells, CD8^+^ T cells, neutrophils, macrophages, and dendritic cells) in GBM. Additionally, the TRRUST database v.2.0 (https://www.grnpedia.org/trrust/) was used to identify transcription factors (TFs) associated with the hub genes, determining critical regulators for these genes [18].

### 2.6 Survival analysis and expression of hub genes

Survival analysis was conducted using Gene Expression Profiling Interactive Analysis 2 (GEPIA2) to assess the relationship between hub gene expression and GBM survival time. RNA sequencing data were integrated from 9736 tumor tissues and 8587 normal tissues from the TCGA and GTEx databases [19]. The Kaplan-Meier plotter was used to classify expression values into high and low groups, with the hazard ratio (HR) calculated to evaluate the relationship between gene expression and survival [20]. Further validation of prognostic analysis was performed using the Chinese Glioma Genome Atlas (CGGA), which includes mRNA sequencing datasets for specific glioma subtypes [21].

### 2.7 Selection of inhibitory compounds targeting hub proteins

Ligand-based virtual screening (VS) was conducted to investigate large datasets of compounds efficiently and cost-effectively [22]. The Naturally Occurring Plant-based Anticancer Compound-Activity-Target (NPACT) database, containing 1574 entries of plant-derived natural compounds with anticancer activity, was utilized [23]. A total of 120 phytochemicals that act against brain cancer were selected for further analysis.

### 2.8 ADMET prediction

The SMILES strings for the 120-brain cancer inhibitory compounds were retrieved from the PubChem database and screened for their ADMET (Absorption, Distribution, Metabolism, Excretion, and Toxicity) properties using the SwissADME tool (http://www.swissadme.ch/). By predicting the compound's behavior in biological systems, ADMET assessment helps lower the risk of clinical trial failure by evaluating compounds based on their drug-like physicochemical and pharmacokinetic features [24]. The principal molecular characteristics utilized in the computation of lead molecule pharmacokinetic features are the partition coefficient (logP), molecular weight (MW), or the counts of donors and acceptors of hydrogen bonds. These characteristics play a part in creating Lipinski's Rule of Five (Ro5) [25], commonly known as the Pfizer rule. We selected compounds predicted to be blood brain barrier permeable (BBB). The final ligands that passed these ADMET properties were selected, and their 3D structures were downloaded in SDF format. The toxicity of the selected compounds was evaluated using pkCSM [26]. The ADME/Tox properties can be predicted quickly and accurately thanks to the pkCSM—pharmacokinetics server (https://biosig.lab.uq.edu.au/pkcsm/). It was constructed using carefully chosen data sets and documented techniques that were found in the literature.

### 2.9 Structure preparation and molecular docking

Molecular docking was used to investigate the atomic-level interactions between proteins and small molecule ligands [27]. The three-dimensional crystal structures of the proteins EGFR, PIK3CA, PTPN11, and KRAS were retrieved from the RCSB Protein Data Bank, using PDB IDs 1M14, 8EXL, 3B7O, and 4EPR, respectively. The ligand binding sites as shown in Table 3 were derived from the published literature [[28], [29], [30], [31]]. The structure of the SRC protein was obtained from the AlphaFold database (AF-P12931-F1). All protein structures underwent geometry optimization and energy minimization using the Dock Prep tool in UCSF Chimera [32], with water molecules and heteroatoms removed and charges and hydrogen atoms added. The finalized protein structures were stored in PDBQT format. The selected phytochemicals were also prepared for docking by downloading their three-dimensional structures from PubChem and minimizing them using UCSF Chimera.

Molecular docking analysis was conducted using AutoDock Vina [33], generating ten conformations of the docked complexes. These were ranked based on binding energy and RMSD values to set up the grid box encompassing the binding sites. Molecular interactions were visualized using BIOVIA Discovery Studio (DS) 2021 client.

### 2.10 Molecular dynamics simulations

Employing the Desmond module from the Schrödinger software suite [34], this investigation performed molecular dynamics (MD) simulations on five specific ligand-protein complexes, aiming to meticulously explore their dynamic interactions and stability within a simulated physiological environment. The complexes under examination were strategically chosen based on their promising interactions revealed in preliminary molecular docking studies, signifying their potential therapeutic relevance against GBM. These complexes encompass Isochaihulactone and Vismione B in conjunction with both EGFR and SRC proteins and Resveratrol with PIK3CA.

The Desmond System Builder tool facilitated the preparation of each ligand-protein complex for simulation. Each complex was placed within an orthorhombic box, generously solvated with a TIP3P water model to ensure a realistic hydration environment. A buffer zone of at least 10 Å was established around the complexes to minimize boundary effects and interactions with the box edges. To emulate biological ion concentration and neutrality, sodium or chloride counterions were added accordingly, neutralizing the overall charge of the simulation system. Following system preparation, the simulations were performed adhering to the NPT ensemble, maintaining constant pressure and temperature throughout. Initial energy minimization was undertaken to resolve any potential steric clashes, paving the way for a seamless progression to the equilibrium phase. Subsequently, the system temperature was gradually elevated to 300K, aligning with physiological conditions. The core MD simulation was extended over a timeframe of 100 ns, offering a detailed view of the temporal evolution of ligand-protein interactions. The duration of the simulation time frame provides us with enough data to evaluate the protein-ligand structural conformation variations and stability later. It was selected based on preliminary tests indicating the necessity for a more extended period to observe meaningful conformational shifts and interaction patterns [35,36].

Post-simulation trajectory analyses were meticulously performed using the Schrödinger Maestro interface, emphasizing the evaluation of the complexes' stability and interaction nuances over the simulation period. Root-mean-square deviation (RMSD) metrics for both protein backbones and ligands were computed frame-by-frame across the entire trajectory of the MD simulation to assess structural stability. The calculations focused on the backbone atoms of the protein and all atoms of the ligand, providing insights into the dynamic stability of each complex. Moreover, detailed inspections of ligand-protein interactions were carried out, focusing on bond formations, interaction strengths, and conformational changes significant to therapeutic efficacy.

### 2.11 MM-GBSA binding energy calculation

In the subsequent phase of the investigation, the Prime MM-GBSA tool within the Schrödinger suite, previously utilized for molecular dynamics (MD) simulations, was employed to accurately assess the binding energies of the phytochemicals Isochaihulactone, Vismione B, and Resveratrol with their target proteins. This analysis was facilitated by the thermal_mmgbsa.py script, known for its in-energy computation, allowing for a comprehensive examination of the interaction dynamics between the ligands and proteins throughout the simulation. To ensure a broad analysis encompassing a range of conformational states, frames were systematically extracted from the MD simulation trajectories at regular intervals, specifically every 10 ps throughout the entire 100ns simulation period [37].

This approach enabled the detailed dissection of the binding energy into several key components, offering a nuanced view of the molecular interactions involved. By evaluating the overall binding energy along with specific contributions from electrostatic, covalent, and hydrogen-bond interactions, as well as lipophilic, pi-pi stacking, solvation, and van der Waals forces, the study provided insights into the complex energetics that govern the affinity of Isochaihulactone, Vismione B and Resveratrol towards their respective targets. Such an exhaustive examination of energy components is crucial in shedding light on the potential of these phytochemicals as inhibitors against glioblastoma, contributing valuable knowledge to the field of drug discovery and development in the fight against this formidable brain cancer.

## 3 Results

### 3.1 Identification of GBM-associated genes

The identification of genes associated with glioblastoma (GBM) was conducted through a comprehensive bioinformatics analysis. Initially, 2473 GBM-associated genes were retrieved from the GeneCards database, and 1707 genes were identified from the DisGeNET database. To refine these findings, two datasets from the Gene Expression Omnibus (GEO) were analyzed: GSE12657, which included 7 GBM samples and 5 normal brain samples analyzed using the GPL8300 [HG_U95Av2] Affymetrix Human Genome U95 Version 2 Array platform, and GSE86202, which comprised 3 GBM samples and 3 normal brain samples analyzed using the GPL16791 Illumina HiSeq 2500 platform (Table 1).

**Table 1:** Information regarding GEO datasets included in this study.

Sample Accession No.	GBM Samples	Control Samples	Sample type	Platform
GSE12657	07	05	human glioma samples	GPL8300
GSE86202	03	03	human glioma samples	GPL16791

**Fig. 2:**
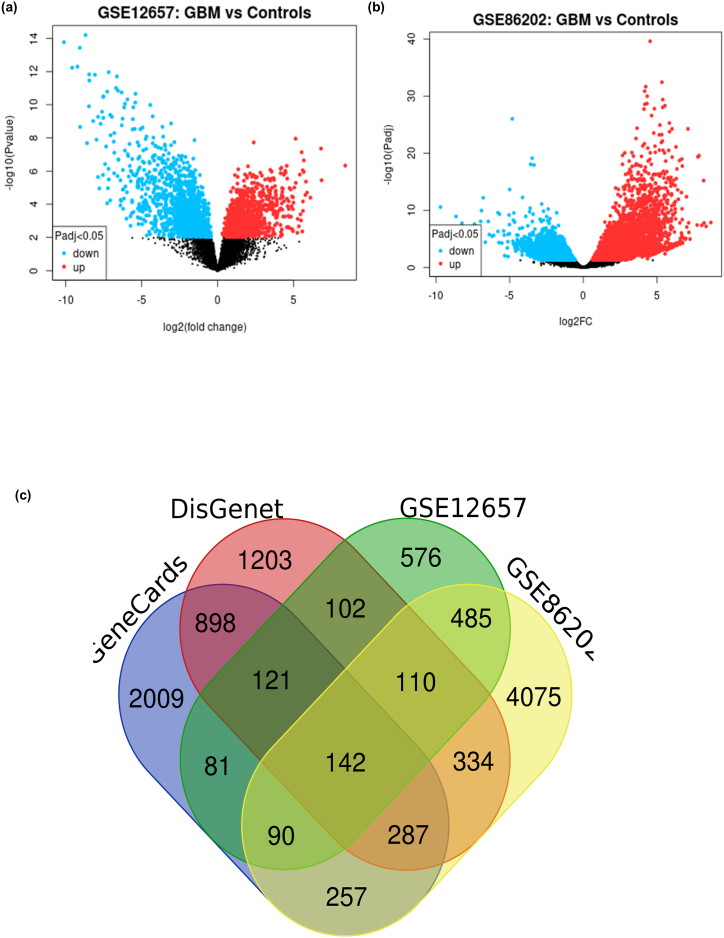
**Differential expression and overlap of GBM-associated genes. (a)** Volcano plot of DEGs in the GSE12657 dataset, comparing GBM tissues to controls; **(b)** Volcano plot of DEGs in the GSE86202 dataset, highlighting upregulated and downregulated genes; **(c)** Venn diagram showing the overlap of GBM-associated genes across GeneCards, DisGeNET, GSE12657, and GSE86202 datasets, with 142 common genes identified.

Differential gene expression analysis between GBM tissues and healthy controls was performed for both datasets, with significant differentially expressed genes (DEGs) identified using thresholds of an adjusted p-value <0.05 and |log2FoldChange (log2FC)| > 1. The DEGs were visualized through volcano plots, which illustrated the distribution of upregulated and downregulated genes in GBM compared to controls (Fig. 2a and b). The GSE12657 dataset showed a clear separation of significantly altered genes, as did the GSE86202 dataset, highlighting the distinct gene expression profiles associated with GBM.

To further pinpoint the most relevant GBM-associated genes, overlapping genes among the GeneCards, DisGeNET, GSE12657, and GSE86202 datasets were identified. A total of 142 genes were found to be common across all four datasets, indicating their potential significance in GBM pathology. These overlapping genes, visualized in a Venn diagram, represent a refined list of candidates that may play critical roles in the development and progression of GBM, thus warranting further investigation (Fig. 2c) (Supplementary Table 1).

### 3.2 GO and KEGG enrichment analysis of core genes

**Fig. 3:**
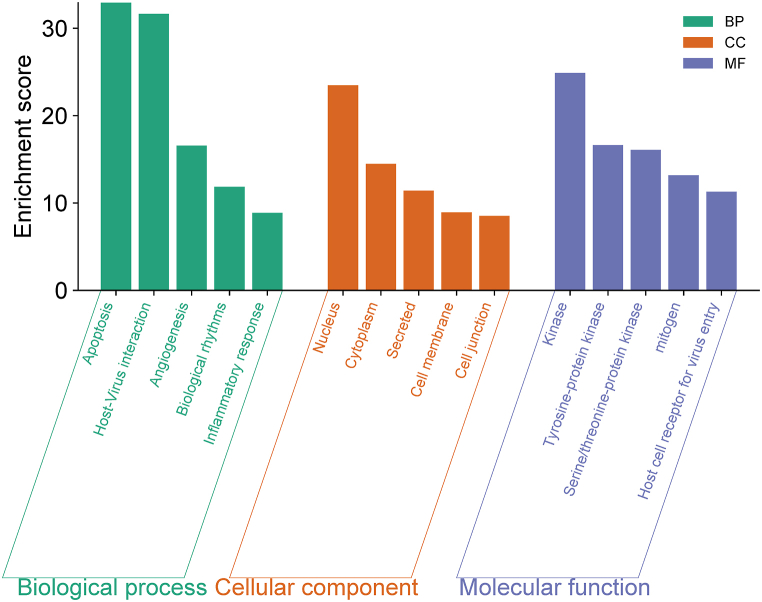
**GO Enrichment Analysis of Core Genes in GBM.** The bar chart displays the top five enriched GO terms for core genes in three categories: Biological Processes (BP), Cellular Components (CC), and Molecular Functions (MF).

Gene Ontology (GO) and Kyoto Encyclopedia of Genes and Genomes (KEGG) enrichment analyses were conducted to elucidate the biological functions of core genes in glioblastoma (GBM) and the regulatory pathways in which they are involved. The GO analysis focused on three categories: Biological Processes (BP), Cellular Components (CC), and Molecular Functions (MF). The top five enrichment results for each GO category are presented in Table 2. The GO enrichment analysis revealed that, among the BP terms, core genes were significantly associated with apoptosis, host-virus interaction, angiogenesis, biological rhythms, and inflammatory responses. In the CC category, the top entries were related to the nucleus, cytoplasm, secreted cellular components, and cell junctions. For MF, the most enriched terms included kinase regulator activity, tyrosine-protein kinase activity, serine/threonine-protein kinase activity, mitogen activity, and host cell receptor activity for virus entry (Fig. 3).

**Table 2:** GO and KEGG enrichment analysis with GO enrichment analysis: the top 5 biological processes (BP), cell components (CC), and molecular functions (MF).

Category	GO Pathways	P-value	Genes counts
Go Biological processes	Apoptosis	1.2E-33	132
	Host-Virus interaction	2.3E-32	147
	Angiogenesis	2.7E-17	46
	Biological rhythms	1.4E-12	41
	Inflammatory response	1.4E-9	42
Cellular Component	Nucleus	3.3E-24	532
	Cytoplasm	3.3E-15	490
	Secreted	4.0E-12	218
	Cell membrane	1.2E-9	342
	Cell junction	3.1E-9	60
Molecular Function	Kinase	1.3E-25	151
	Tyrosine-protein kinase	2.4E-17	43
	Serine/threonine-protein kinase	8.4E-17	86
	mitogen	6.6E-14	23
	Host cell receptor for virus entry	5.3E-12	28
KEGG Pathway disease terms	MicroRNAs in cancer	7.6E-83	172
	Pathways in cancer	7.8E-80	225
	Proteoglycans in cancer	4.1E-52	112
	PI3K-Akt signaling pathways	1.7E-48	147
	Hepatitis B	1.1E-47	95

**Table 3:** Docking results for selected phytochemicals with GBM-Associated hub proteins.

Genes Name	PDB ID	Proteins Name	Binding/Active sites	Ligand (PubChem ID)	RMSD (A°)	Binding Energies (KJ/Mol)	H-bond distance (A°)	H-bond interaction
EGFR	1M14	Tyrosine Kinase Domain from Epidermal Growth Factor Receptor	Leu 718, Gly 719, Ser 720 Gly 721, Ala 722, Phe 723 Gly 724, Thr 725, Val 726 Lys 745, Met 790, Gln 791 Asp 837, Asp 855	Isochaihulactone (641765)	1.628	−7.546	2.70	Ala 698
				Vismione B (10247551)	1.580	−6.441	3.48, 3.81	Thr 766, Asp 831
PIK3CA	8EXL	Crystal structure of PI3K-alpha in complex with taselisib	Met772, Ser774, Pro778, Met800, Lys802, Asp810, Tyr836, Ile848-Val851, Met922, Ile932, Asp933	Resveratrol (445154)	1.121	−6.032	5.21, 4.51	Ser 774, Asp 933
SRC	Alpha-fold (AF-P12931-F1)	Proto-oncogene tyrosine-protein kinase Src	Lue 276, Gly 277, Gln 278, Gly 279, Cys 280, Phe 281 Gly 282, Glu 283, Val 284 Lys 298, Asp 389	Isochaihulactone (641765)	1.617	−6.963	5.38, 6.40	Lys 298, Arg 391
				Vismione B (10247551)	0.973	−6.375	5.36, 5.89	Lys 298, Arg 391

**Fig. 4:**
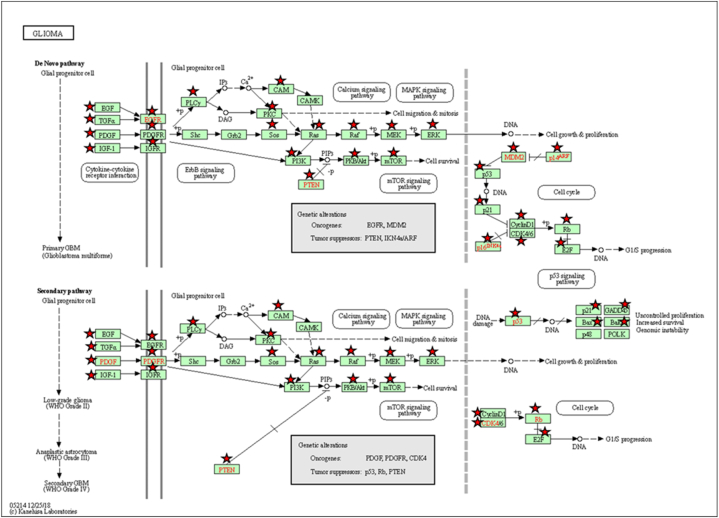
**KEGG Pathway Enrichment Analysis Highlighting the Glioma Pathway.** The diagram illustrates the critical KEGG pathways in which the identified core genes are involved, with a focus on the Glioma pathway.

In addition to the GO terms, several significant KEGG pathways (p-value <0.05) were identified, with the most prominent being microRNAs in cancer, pathways in cancer, proteoglycans in cancer, PI3K-Akt signaling pathways, and Hepatitis B. Notably, the Glioma pathway, which includes 50 core genes, was highlighted with a Benjamini-Hochberg corrected p-value of 1.1E-27, emphasizing its relevance to GBM. These findings are depicted in Fig. 4, illustrating the critical pathways in which these core genes are involved.

### 3.3 PPI network visualization and hub gene identification

**Fig. 5:**
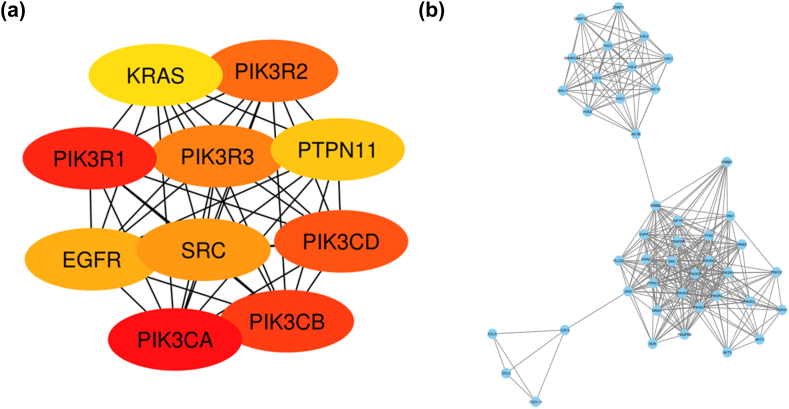
**PPI Network Visualization and Hub Gene Identification. (a)** The top 10 hub genes identified using the Maximal Clique Centrality (MCC) algorithm in the 'cytoHubba' plugin are visualized; **(b)** The application of the MCODE algorithm in Cytoscape identified 33 clusters within the PPI network.

To explore the interactions among the 1412 overlapping genes, a protein-protein interaction (PPI) network was constructed using the STRING online database. The analysis focused on the highest confidence target protein interaction network, with a confidence score of 0.9, resulting in a network comprising 1209 nodes and 5414 edges, with a PPI enrichment p-value of <1.0E-16. The resulting PPI network file was then imported into Cytoscape 3.9.1 for further analysis. Within this network, the top 10 core nodes were identified and ranked using the Maximal Clique Centrality (MCC) algorithm in the ‘cytoHubba’ plugin. The genes identified as hub genes were PIK3CA, PIK3R1, PIK3CB, PIK3CD, PIK3R2, PIK3R3, SRC, EGFR, PTPN11, and KRAS (Fig. 5a). Additionally, by applying the MCODE application in Cytoscape, 33 clusters were identified within the network. The top-ranked cluster contained 44 nodes, 323 edges, and had an MCODE score of 15.023 (Fig. 5b).

### 3.4 Immune infiltration and prediction of TF-hub genes

The analysis of immune cell infiltration revealed that the identified hub genes were significantly correlated with six types of immune cells, including B cells, CD8^+^ T cells, CD4^+^ T cells, macrophages, neutrophils, and dendritic cells, with varying degrees of association, as determined using the TIMER database (Supplementary Figure 1). Furthermore, the investigation of transcription factor (TF) regulatory relationships via the TRRUST database identified the Sp1 transcription factor (SP1) as a key regulator. SP1 was found to directly interact with EGFR and SRC proteins, with a statistically significant p-value of 0.024.

### 3.5 Survival Analysis and Expression Levels of Hub Genes

**Fig. 6:**
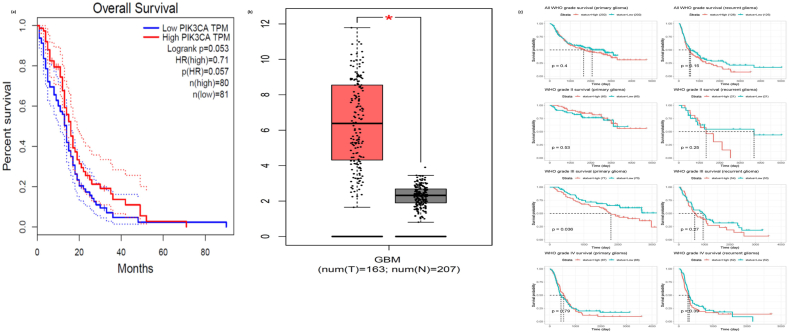
**Survival Analysis and Expression Levels of Hub Genes in GBM. (a)** Kaplan-Meier survival curves for PIK3CA, showing a significant correlation between higher PIK3CA expression and worse overall survival (OS) in GBM patients; **(b)** Expression levels of EGFR, significantly elevated in tumor tissues compared to normal tissues; **(c)** Validation of PIK3CA's significance in WHO-grade gliomas using the CGGA database, confirming its association with poor OS.

Kaplan-Meier survival analysis was performed on the top 10 ranked hub genes using the GEPIA2 database. Among these, PIK3CA was found to be statistically significant (P ≤ 0.05), with higher expression levels of PIK3CA (HR 0.05, P = 0.05) correlating with worse overall survival (OS) in GBM patients (Fig. 6a). This suggests that PIK3CA could serve as a potential biomarker for monitoring GBM progression. Additionally, the expression level of EGFR was significantly different between normal and tumor tissues (P ≤ 0.05), with a marked increase in expression observed in tumor samples (Fig. 6b). These findings were further validated using the CGGA database, which also showed statistical significance for the PIK3CA gene (P = 0.036) in WHO-grade gliomas, confirming its association with poor OS in GBM patients (Fig. 6c).

### 3.6 Virtual screening of the NPACT database

The NPACT database, which focuses on anti-cancerous natural molecules derived from plants, was utilized to identify potential inhibitors for brain cancer. A total of 120 phytochemicals from the database were initially selected for their inhibitory functions in brain cancer. Given the importance of ADME (Absorption, Distribution, Metabolism, and Excretion) properties in drug development, only compounds that met the necessary ADME requirements were considered further. Out of these 120, 40 compounds passed ADME filtering. Subsequently, only those compounds that demonstrated the potential to cross the blood-brain barrier (BBB) were retained, resulting in a final selection of 10 compounds with favorable brain distribution potential (Supplementary Fig. 2).

Drug-likeness analysis of these compounds showed no violations of Lipinski's Rule of Five (Ro5), with all compounds demonstrating good oral bioavailability, as indicated by values ranging from 0.55 to 0.56. These compounds also exhibited promising pharmacokinetic properties, lacked toxic effects, and displayed overall good drug-like characteristics (Supplementary Table 2). Further analysis using ADMET tests for hepatotoxicity, endocrine system toxicity, and neurotoxicity identified Isochaihulactone and Oropheolide as having no predicted toxic risks to liver function, the endocrine system, or the reproductive system (Supplementary Table 3).

### 3.7 Docking of the target compounds with top hub genes

The docking analysis aimed to evaluate the binding efficiency of the 10 selected phytochemicals, which were identified for their ability to effectively pass the BBB, with the top 10 hub genes, focusing on their capacity to bind at the active sites. The structures of these compounds are shown in Supplementary Table 4. We selected poses with binding energies below −6 kJ/mol and identified key interactions, preferably hydrogen bonds, with active site residues between the ligand and receptor to determine successful binding. This screening approach enables us to identify poses that are more likely to represent biologically relevant binding conformations. Among the tested compounds, Isochaihulactone and Vismione B demonstrated high-affinity binding pose to their target regions, specifically interacting with the hub genes EGFR and SRC.

Isochaihulactone exhibited strong binding to the Tyrosine Kinase Domain of EGFR (PDB ID: 1M14), with a binding energy of −7.546 kJ/mol. It formed a conventional hydrogen bond with Ala698, with a bond distance of 2.70 Å. Additionally, Isochaihulactone also interacted with SRC (AlphaFold model, AF-P12931-F1), binding to the active site residues Lys298 and Arg391, with a binding energy of −6.963 kJ/mol, forming hydrogen bonds with distances of 5.38 Å and 6.40 Å, respectively.

Vismione B also showed effective binding to EGFR and SRC. For EGFR, Vismione B bound to Thr766 and Asp831 with binding energies of −6.441 kJ/mol, forming hydrogen bonds with distances of 3.48 Å and 3.81 Å. In SRC, Vismione B interacted with Lys298 and Arg391, with a binding energy of −6.375 kJ/mol, forming hydrogen bonds with distances of 5.36 Å and 5.89 Å.

**Fig. 7:**
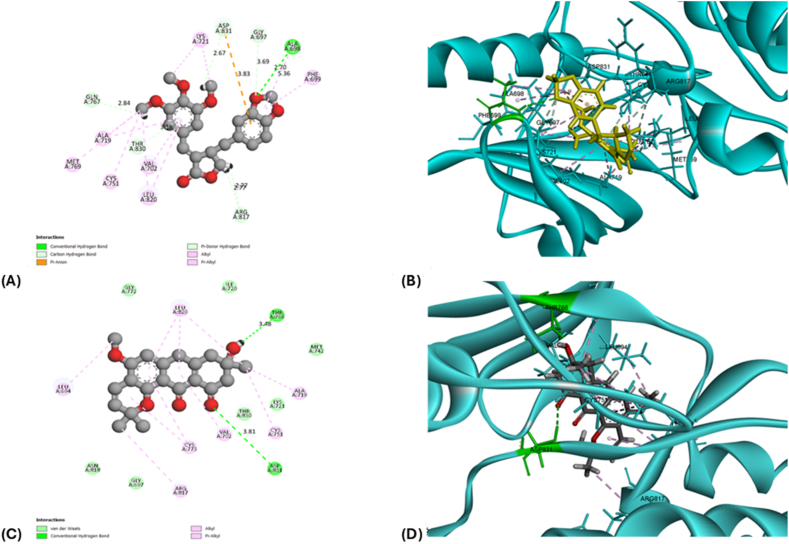
**The 3-dimensional and 2-dimensional interaction of EGFR with their target ligands**. A) The 2-D interaction of EGFR with Isochaihulactone. B) The 3-D interaction of EGFR with Isochaihulactone. C) The 2-D interaction of EGFR with VismioneB. D) The 3-D interaction of EGFR with VismioneB.

**Fig. 8:**
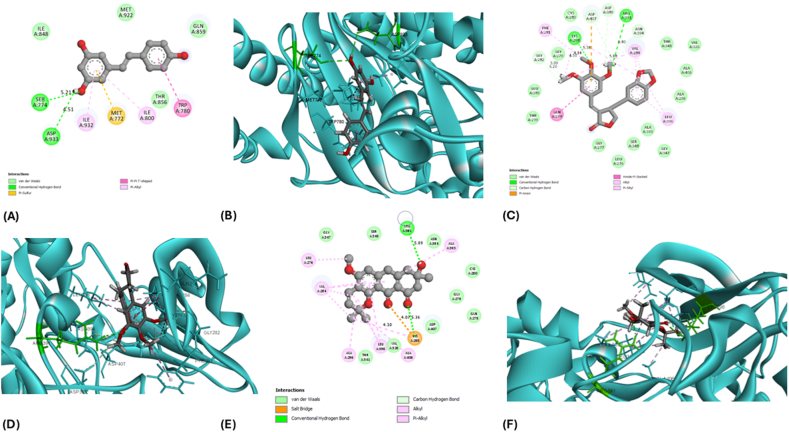
**The 3-dimensional and 2-dimensional interaction of PIK3CA and SRC with their target ligands**. A) The 2-D interaction of PIK3CA with Resveratrol. B) The 3-D interaction of PIK3CA with Resveratrol. C) The 2-D interaction of SRC with Isochaihulactone. D) The 3-D interaction of EGFR with Isochaihulactone. E) The 2-D interaction of SRC with VismioneB, F) The 3-D interaction of SRC with VismioneB.

Additionally, Resveratrol exhibited binding potential with PIK3CA (PDB ID: 8EXL), interacting with the active site residues Ser774 and Asp933. The binding energy was recorded at −6.032 kJ/mol, forming hydrogen bonds with distances of 5.21 Å and 4.51 Å. The detailed docking scores, including binding energies and RMSD values, are provided in Table 3. Visual representations of these interactions are illustrated in Fig. 7, Fig. 8. These interactions indicate strong and specific binding of these phytochemicals to their respective target proteins. Hence, these five complexes—Isochaihulactone with EGFR and SRC, Vismione B with EGFR and SRC, and Resveratrol with PIK3CA—were further analyzed using molecular dynamics (MD) simulations and MM-GBSA to assess their stability and binding energy profiles in a dynamic environment.

### 3.8 Molecular dynamics simulations

**Fig. 9:**
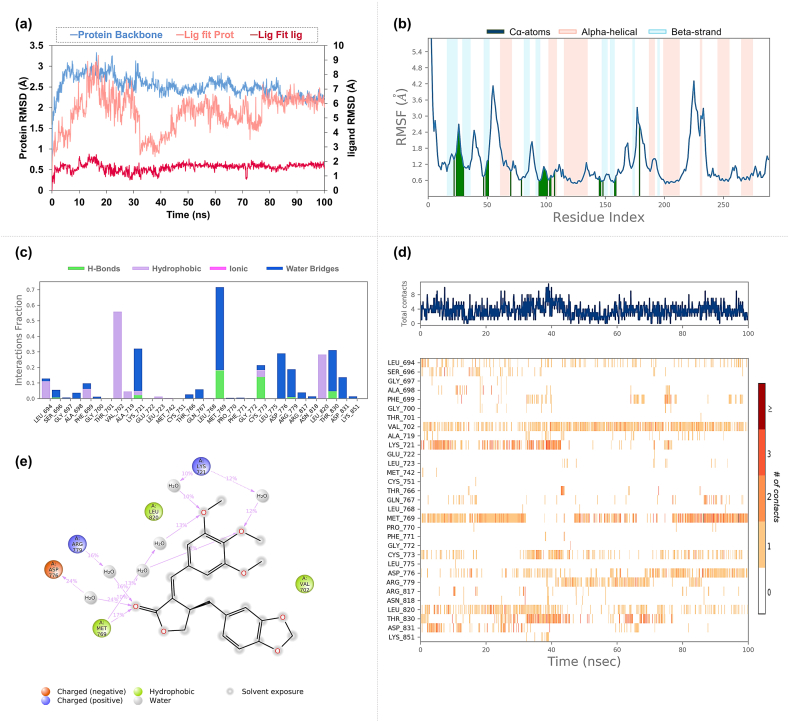
**Molecular Dynamics Simulation Profiles of Isochaihulactone Docked in EGFR** (a) The RMSD (Root Mean Square Deviation) profile, illustrating the stability of the protein-ligand complex over the simulation time. (b) The RMSF (Root Mean Square Fluctuation) profile, depicts the flexibility of protein residues throughout the course of the simulation. (c) The interaction fraction profile shows the percentage of simulation time that each amino acid within the binding site is involved in interactions with the ligand. (d) The interaction timeline highlights the persistence and duration of contacts between the ligand and specific amino acids in the binding pocket. (e) The detailed schematic of the types of interactions, such as hydrogen bonds, hydrophobic interactions, and Pi interactions, occurring between Isochaihulactone and EGFR during the simulation.

In the Molecular Dynamics (MD) simulations of the EGFR-Isochaihulactone complex, a moderate fluctuation in the backbone of protein was observed with an RMSD mean value of 2.52 Å (Fig. 9a). Isochaihulactone exhibited notable stability (5.4 Å) when bound to the protein, although individual ligand stability showed a lower degree of fluctuation (1.6 Å). The RMSF analysis indicated variable flexibility along the EGFR chain, with the most significant movements occurring in loop regions, as highlighted in Fig. 9b. The interaction analysis demonstrated that Isochaihulactone forms stable hydrogen bonds with key EGFR residues, including Lys721, Met769, Cys773, and Thr830, as well as hydrophobic interactions with Val702, as detailed in Fig. 9c–e.

**Fig. 10:**
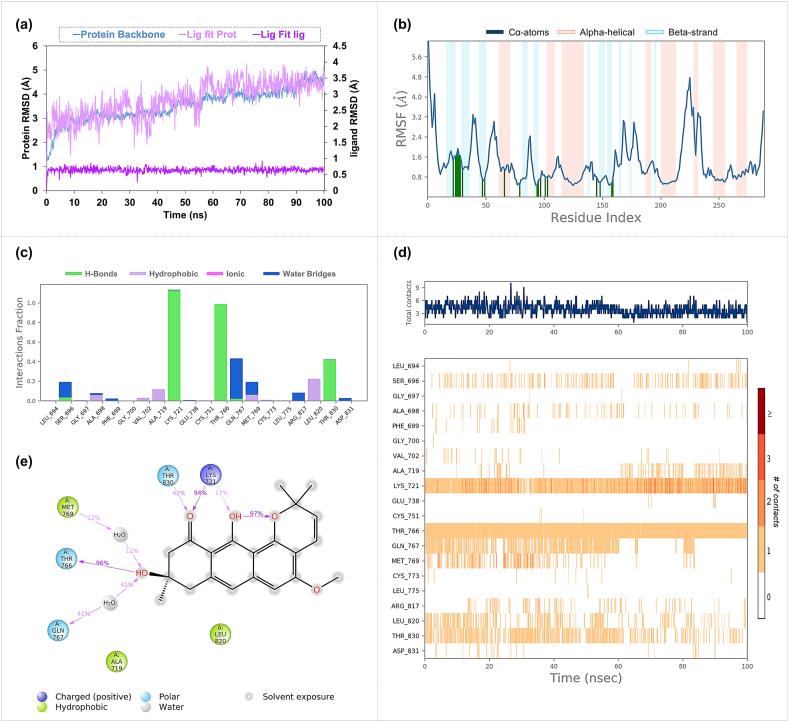
**Molecular Dynamics Simulation Profiles of Vismione B Docked in EGFR** (a) Displays the RMSD profile, indicating the overall stability of the EGFR-Vismione B complex over time. (b) Shows the RMSF profile, which maps out the fluctuation of each residue in EGFR, revealing areas of flexibility and rigidity. (c) Presents the amino acid interaction fraction, quantifying the proportion of time that each amino acid in the active site engages with Vismione B. (d) Details the timeline of interaction, documenting the consistency and time-specific interactions between Vismione B and EGFR residues. (e) Describes the types of interactions, including hydrogen bonds and hydrophobic contacts, formed between the ligand Vismione B and the protein EGFR.

The EGFR-Vismione B complex showed a stable protein conformation with an average RMSD of 3.54 Å for the protein backbone (Fig. 10a). The ligand was particularly stable, evidenced by low RMSD (2.83 Å). RMSF results for this complex (Fig. 10b) showed a similar pattern of flexibility to the Isochaihulactone complex, with binding site interactions delineated by green bars. Hydrogen bonds were consistently formed between Vismione B and the EGFR residues Lys721, Thr776, and Thr830. Additionally, Gln767 and Met769 were identified as significant contributors to the stability of the ligand within the active site through water bridge interactions, as presented in Fig. 10c–e.

**Fig. 11:**
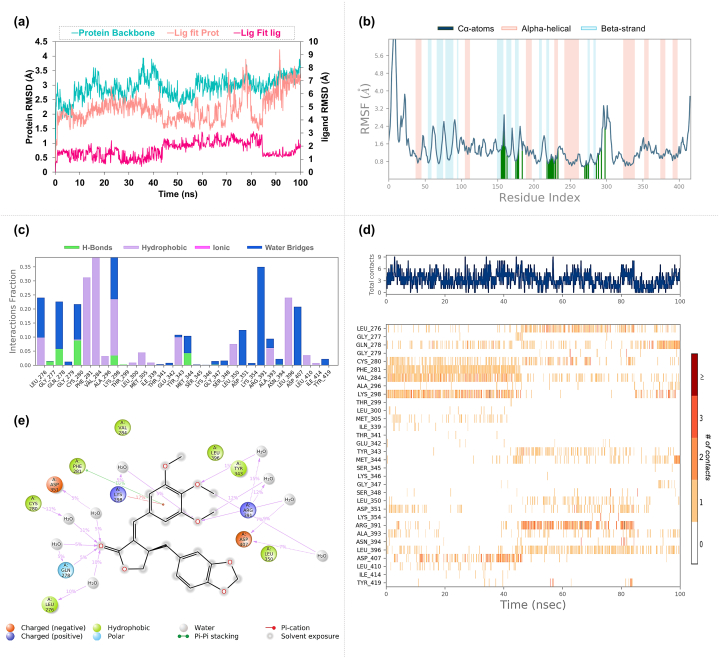
**Molecular Dynamics Simulation Profiles of Isochaihulactone Docked in SRC** (a) The RMSD profile for the SRC-Isochaihulactone complex, assessing the stability across the simulation timeframe. (b) The RMSF profile provides a detailed view of the SRC protein residues' flexibility during the interaction with Isochaihulactone. (c) The interaction fraction of amino acids reflects the percentage of time each amino acid in SRC interacts with Isochaihulactone. (d) The interaction timeline, outlining the sustained and transient contacts of Isochaihulactone with SRC residues. (e) The schematic details the interaction types, highlighting hydrogen bonds, water bridges, and other relevant interactions between Isochaihulactone and SRC.

The MD simulations of the SRC-Isochaihulactone complex indicated a protein backbone RMSD mean value of 2.93 Å, with the ligand demonstrating considerable mobility when fitted to the protein (Fig. 11a). The ligand alone showed an RMSD mean value of 1.71 Å, indicating relative stability. RMSF analysis indicated that the interaction of amino acids within the SRC binding site exhibited lower fluctuations (Fig. 11b). However, interaction analysis revealed that Isochaihulactone maintained a less stable interaction within the SRC binding site, with no amino acid demonstrating more than a 20 % interaction fraction. Notably, Lys298 showed a 17 % interaction through Pi-cation interactions (Fig. 11c–e).

**Fig. 12:**
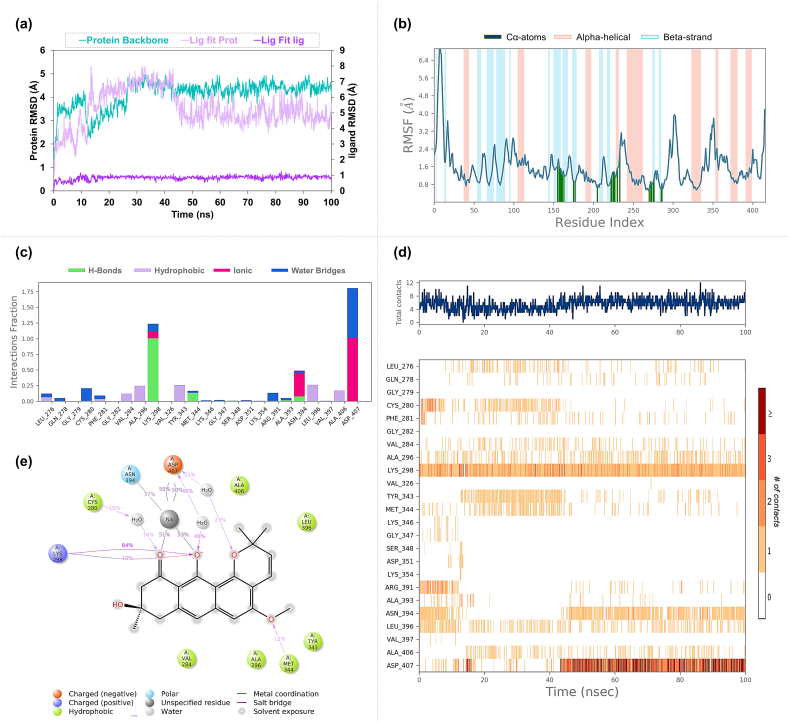
**Molecular Dynamics Simulation Profiles of Vismione B Docked in SRC** (a) Illustrates the RMSD profile for SRC-Vismione B, revealing the stability of this complex throughout the simulation period. (b) Maps out the RMSF profile for SRC, showing fluctuations and the relative mobility of residues when bound to Vismione B. (c) Depicts the interaction fraction, indicating how often each amino acid residue is involved in the interaction with Vismione B. (d) Captures the detailed interaction timeline, reflecting the duration and strength of the binding between Vismione B and SRC residues. (e) Enumerates the types of interactions, such as hydrogen bonds, water-mediated interactions, and pi-stacking, occurring within the SRC-Vismione B complex.

For the SRC-Vismione B complex, the RMSD results reflected greater movement, with a protein backbone mean of 4.12 Å and ligand mean of 0.84 Å, suggesting that the ligand was more stably anchored within the binding pocket (Fig. 12a). Consistent with the Isochaihulactone complex, RMSF values showed that residues involved in binding displayed lower degrees of movement (Fig. 12b). Detailed interaction analysis revealed that Vismione B formed hydrogen bonds with Lys298 and Met344. Additionally, Cys280 and Asp407 were involved in water-mediated interactions within the active site (Fig. 12c–e).

**Fig. 13:**
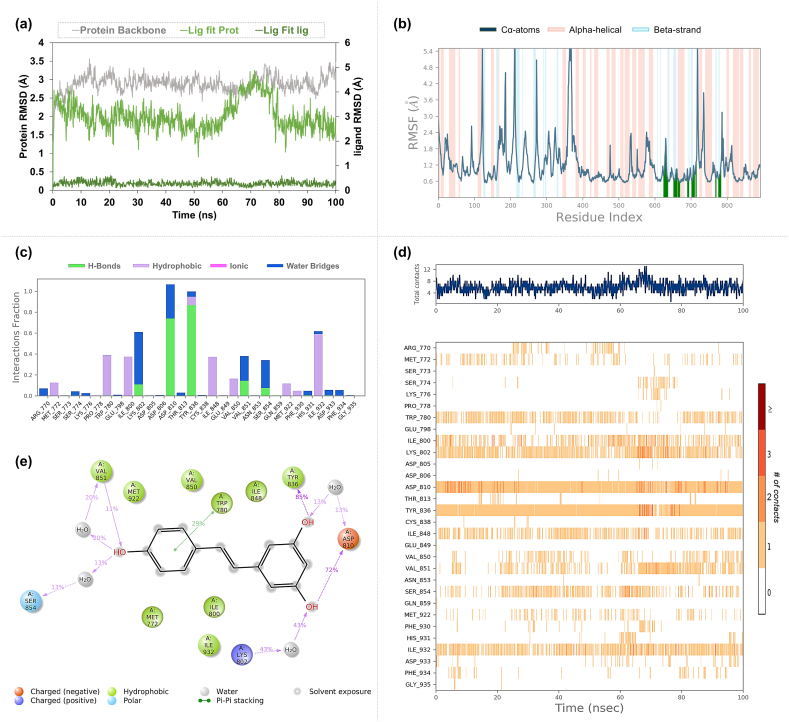
**Molecular Dynamics Simulation Profiles of Resveratrol Docked in PIK3CA** (a) Showcases the RMSD profile for the PIK3CA-Resveratrol complex, evaluating the molecular stability during the simulation. (b) Features the RMSF profile, which analyzes the residue flexibility of PIK3CA in the presence of Resveratrol. (c) Displays the interaction fraction profile for amino acids, presenting the extent of interactions between Resveratrol and the active site residues of PIK3CA. (d) Provides the interaction timeline, indicating the persistent interactions of Resveratrol with PIK3CA throughout the simulation. (e) Details the types of interactions, illustrating the direct hydrogen bonding and water bridge interactions that Resveratrol forms with PIK3CA.

In the PIK3CA-Resveratrol complex, the RMSD results indicated a mean value of 2.88 Å for the protein backbone, suggesting a stable tertiary structure throughout the simulation (Fig. 13a). When examining the ligand's fit to the protein, the RMSD mean was slightly higher at 3.02 Å. In comparison, the ligand's intrinsic RMSD mean was remarkably low at 0.28 Å, denoting significant stability of Resveratrol within the binding site. The standard deviations for these measurements were within acceptable limits, indicating consistent behavior throughout the simulation. The RMSF results were consistent with those observed in the previous complexes, demonstrating the same flexibility patterns along the protein chain (Fig. 13b). The interaction profile detailed in Fig. 13c–e highlighted that Resveratrol formed hydrogen bonds with key residues, including Lys802, Asp810, Tyr836, Val851, and Ser854. Notably, Tyr836 maintained a remarkably stable interaction with an 83 % interaction fraction through direct hydrogen bonds, followed by Asp810 with a 72 % interaction fraction. Water bridges were observed contributing to the interactions with Lys802 and Ser854. At the same time, Trp780 was involved in Pi-Pi stacking interactions, indicating a multifaceted interaction landscape that contributes to the binding affinity and specificity of Resveratrol to the PIK3CA protein.

### 3.9 MM-GBSA analysis

In a comparative analysis of ligand stability, the MM-GBSA results indicated a differential binding affinity of Isochaihulactone and Vismione B with EGFR and SRC proteins (Table 4). The EGFR-Isochaihulactone complex presented a binding free energy of −45.14 ± 6.65 kcal/mol, suggesting a significant affinity, while the EGFR-Vismione B complex displayed a slightly more favorable binding affinity with an average ΔG of −50.05 ± 3.86 kcal/mol. This suggests Vismione B forms a more energetically favorable interaction with the EGFR protein than Isochaihulactone. On the other hand, when interacting with the SRC protein, Isochaihulactone demonstrated a stronger binding affinity (−49.43 ± 6.60 kcal/mol) than it did with EGFR, despite the ligand's increased mobility within the binding pocket. Vismione B showed notably lower binding affinity to SRC (−24.13 ± 5.54 kcal/mol) than EGFR. This substantial difference in binding energies indicates that while Isochaihulactone maintains a relatively consistent binding profile across both proteins, Vismione B prefers EGFR over SRC regarding binding stability.

**Table 4:** Summary of binding energies and interaction energy components for ligands interacting with EGFR, SRC, and PIK3CA.

Ligand	RMSD (Å)	ΔGBind (Kcal/mol)	ΔGCoulomb (Kcal/mol)	ΔGHbond (Kcal/mol)	ΔGLipo (Kcal/mol)	ΔGPacking (Kcal/mol)	ΔGvdW (Kcal/mol)
**Ligands interacting with EGFR**							
Isochaihulactone	5.398	−45.144	−11.105	1.015	−0.300	−14.211	−40.510
Vismione B	2.830	−46.926	−17.846	3.698	−1.676	−11.408	−45.350
**Ligands interacting with SRC**							
Isochaihulactone	4.886	−41.356	−8.432	2.483	−0.287	−14.262	−39.818
Vismione B	5.282	−24.128	−87.662	5.022	−1.526	−7.896	−33.947
**Ligands interacting with PIK3CA**							
Resveratrol	3.019	−44.885	−18.943	1.966	−1.557	−21.083	−30.573

**Note, meaning of abbreviations used in the table are as follows**:

Coulomb—Coulomb energy.

Hbond—Hydrogen-bonding correction.

Lipo—Lipophilic energy.

Packing—Pi-Pi packing correction.

vdW—Van der Waals energy.

**Fig. 14:**
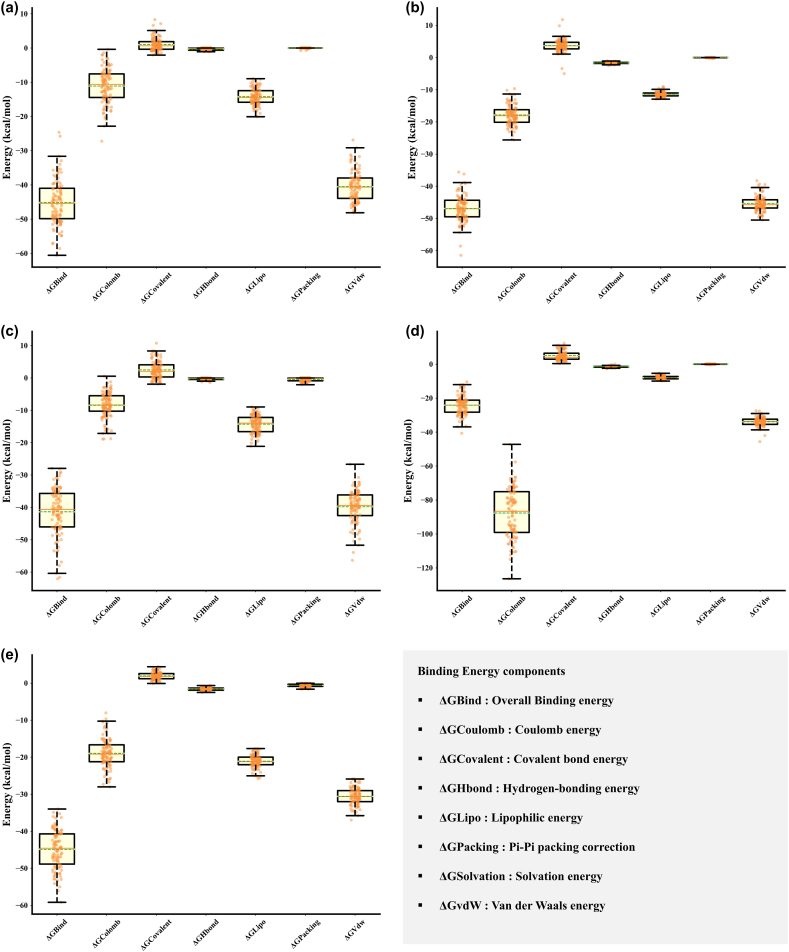
**Analysis of MM-GBSA Binding Energy Components Boxplots display the distribution of MM-GBSA binding energy components for each ligand-protein complex** (a) EGFR-Isochaihulactone Complex, (b) EGFR-Vismione B Complex, (c) SRC-Isochaihulactone Complex, (d) SRC-Vismione B Complex, (e) PIK3CA-Resveratrol Complex. Each boxplot outlines the median (central line), interquartile range (box edges), and 1.5x interquartile range (whiskers), with outliers as individual points.

For the PIK3CA-Resveratrol complex, the MM-GBSA binding free energy was calculated to be −44.88 ± 5.28 kcal/mol. This places Resveratrol's binding affinity in a similar range to that of Isochaihulactone with EGFR and SRC, suggesting that Resveratrol binds with a comparable level of stability to PIK3CA as Isochaihulactone does to its target proteins. The MM-GBSA binding energy components, as delineated in Fig. 14(a–e), were integral in the evaluation of the binding free energies for the interactions between the ligands Isochaihulactone, Vismione B, and Resveratrol with the proteins EGFR, SRC, and PIK3CA, respectively. The box plots in Fig. 14 visually represent the energy distribution, clearly showing the spread and central tendency of each component's contribution to the overall binding energy. These components encompass the overall binding energy (ΔG Bind), Coulomb energy (ΔG Coulomb), covalent bond energy (ΔG Covalent), hydrogen-bonding energy (ΔG Hbond), lipophilic energy (ΔG Lipo), pi-pi packing correction (ΔG Packing), solvation energy (ΔG Solvation), and van der Waals energy (ΔG vdW).

The analysis revealed that, while all the ligands demonstrated favorable binding energies to their respective targets, there were variances in the contribution of individual energy components to the total binding free energy. The ΔG Covalent, ΔG Hbond, and ΔG vdW consistently provided major contributions to the binding process, with other components like ΔG Coulomb exhibiting more variability. This variability indicates the dynamic and sensitive nature of the electrostatic interactions that the spatial orientation of charged amino acid side chains within the binding pockets of the target proteins can influence. These findings from the MM-GBSA energy component analysis are critical as they underscore the complexities of ligand-protein interactions and support the potential of the studied ligands as inhibitors for therapeutic interventions.

## 4 Discussion

Glioblastoma (GBM) is one of the most aggressive and lethal brain tumors, characterized by a high proliferation rate and extensive vascularization, making it particularly challenging to treat [38,39]. Traditional chemotherapeutic and radiation treatments often carry the risk of inducing mutations in healthy cells, potentially leading to secondary malignancies. In contrast, certain natural compounds, particularly flavonoids and polysaccharides, have gained attention for their potential to mitigate disease processes while offering advantages such as lower production costs, wide availability, and better patient compliance [40,41]. However, it is crucial to recognize that generalizing these specific compounds to all-natural compounds may be inappropriate, as the efficacy and safety profiles can vary significantly among different substances. Further research is needed to validate these benefits across a broader range of natural compounds [42,43]. This research aimed to perform virtual screening to identify the most promising compounds within the NPACT database with the potential to inhibit key GBM-associated hub proteins.

The findings of this study suggest that out of the 120 phytochemicals analyzed from the NPACT database, ten were identified as capable of crossing the blood-brain barrier (BBB) and meeting all of Lipinski's Rule of Five criteria, indicating good drug-likeness properties. Further bioinformatics analysis revealed that these phytochemicals predominantly target genes associated with microRNAs in cancer, pathways in cancer, and proteoglycans in cancer pathways. The hub genes identified in this study—PIK3CA, EGFR, and SRC—played critical roles in the prognosis and progression of GBM, suggesting that these genes could serve as critical targets for therapeutic intervention. Kaplan-Meier survival analysis underscored the significance of PIK3CA, as its expression levels were strongly correlated with overall survival in GBM patients, marking it as a potential prognostic biomarker. Additionally, EGFR expression was notably higher in GBM tissues than controls, reaffirming its importance in GBM pathology.

Molecular docking analysis revealed that the phytochemicals Isochaihulactone and Vismione B have the potential to bind to the active site residues of the EGFR and SRC proteins, while Resveratrol binds to key residues of PIK3CA. Further investigation into the stability of these protein-ligand complexes was conducted using molecular dynamics (MD) simulations and MM-GBSA. This approach enabled a detailed dissection of the binding energy into several key components, offering a nuanced view of the molecular interactions involved by evaluating the overall binding energy and specific contributions from electrostatic, covalent, hydrogen-bond interactions, lipophilic, pi-pi stacking, solvation, and van der Waals forces. Both simulations and binding energy calculations showed that while the binding profile of Isochaihulactone remains mostly unchanged for both SRC and EGFR proteins, Vismione B exhibits a preference for EGFR over SRC in terms of stable hydrogen bonds and greater binding stability. The MM-GBSA ΔG values calculations for these compounds demonstrated it and accurately ranked the potential ligands. Vismione B, being top-ranked as the target compound for EGFR, has a slightly better ΔGBind value of −46.926 (Kcal/mol) when compared with Isochaihulactone, while Isochaihulactone is suggested to be a candidate compound when the target is SRC with a ΔGBind value of −41.356 (Kcal/mol). Resveratrol binds to PIK3CA with stability similar to that of Isochaihulactone's binding to its target proteins. The study provided insights into the complex energetics that govern the affinity of Isochaihulactone, Vismione B, and Resveratrol toward their respective targets. Such an exhaustive examination of energy components is crucial in shedding light on the potential of these phytochemicals as inhibitors against glioblastoma, contributing valuable knowledge to drug discovery and development in the fight against this formidable brain cancer.

Isochaihulactone (ICL) is a dibenzylbutyrolactone lignan known for its wide range of pharmacological activities, particularly in cancer treatment. ICL induces cell cycle arrest and promotes apoptosis in cancer cells through various mechanisms. It possesses anti-proliferative and anti-chemoresistance properties, partly by reducing oxidative stress through decreased reactive oxygen species (ROS) production and lipid peroxidation, as well as by maintaining endogenous antioxidant enzyme activities. Furthermore, ICL stabilizes mitochondrial function, which helps attenuate injury in nPC12 cells [44,45]. As a microtubule-depolymerizing agent, ICL disrupts normal cell cycle progression, leading to the inappropriate expression of cyclin B1/cdc2 kinase and Bcl-2 phosphorylation, ultimately triggering the apoptotic cascade. Notably, ICL has demonstrated effectiveness in inhibiting the growth of various tumor cells, including drug-resistant cell lines [46]. Previous studies have reported that Isochaihulactone induces cytotoxicity in various cancer cell lines, including those from lung, breast, ovary, colon, and liver tumors [47].

Vismione B is a cytotoxic naphthopyran compound extracted from the plant *Vismia baccifera* [48]. Species of *Vismia* have been used to treat a wide range of skin conditions, including eczema, dermatitis, leprosy, syphilis, and herpes [49]. Vismiones, which contain a tetrahydroanthracene nucleus, have been isolated from various *Vismia* species and previously reported to exhibit cytotoxic and antitumor activity. These include activity against 9 KB cells in vitro, as well as M5076 ovarian carcinoma and B16 melanocarcinoma transplanted in vivo in experimental animals (mice) [50]. Previous studies have also shown that Vismione B exhibits activity against human breast, central nervous system, and lung cancer cell lines [51]. Resveratrol is a natural polyphenol with known anticancer and chemopreventive properties. It has been demonstrated that resveratrol can reach sub-micromolar concentrations in the brain parenchyma when administered through standard routes, where it inhibits tumor growth through STAT-3-dependent mechanisms. Preclinical data supports the use of resveratrol as an adjuvant in anti-GBM treatment [52].

These results are consistent with previous studies that have identified EGFR as a key prognostic marker and therapeutic target in GBM, with numerous clinical trials investigating EGFR-targeted therapies [53,54]. For instance, a clinical trial involving patients treated after GBM resection with the EGFRvIII vaccine (Rindopepimut) CDX-110 demonstrated longer overall survival [55]. Similarly, erlotinib (OSI-774, CP-358,774, TarcevaTM) is an EGFRK-specific natural inhibitor targeting the kinase domain of the protein and is currently in Phase III clinical trials [56]. It has been reported that 30–40 % of GBMs have an amplified EGFR gene, and nearly 50 % overexpress the receptor. Additionally, EGFR mRNA levels have been detected in less malignant astrocytomas and oligodendrogliomas, even in the absence of gene amplification [57]. Another significant gene identified in this study is PIK3CA, which encodes the p110α catalytic subunit of PI3K, a critical component of the PI3K/Akt pathway [58]. Most human glioblastomas exhibit abnormal activation of PI3K/Akt signalling, either through mutations or amplification in various human neoplasms. This dysregulation is believed to play a significant role in several key characteristics of these highly malignant brain tumors, including disrupted cell cycle regulation, increased resistance to apoptosis, enhanced cell migration and invasion, and abnormal formation of new blood vessels (neo-angiogenesis) [59,60]. There has been a lot of interest in using small molecule inhibitors that target the phosphatidylinositol 3-kinase (PI3K) signaling system to treat cancer. The catalytic component of PI3Kα, the mutant p110α protein, is selectively degraded by a class of benzodiazepine-oxazolidinone ATP-competitive inhibitors of PI3Kα that have been optimized and characterized in recent investigations [61].

The third significant protein of interest is SRC, one of the nine members of the Src family of tyrosine kinases (SFKs), which is implicated in the development and progression of multiple cancer types [62]. The causal connection between Src dysregulation and the cancer phenotype is partially explained by the downstream activation of the phosphatidylinositol 3-kinase (PI3K) and mitogen-activated protein kinase (MAPK) pathways. These pivotal pathways drive critical processes such as proliferation, invasion, tumorigenesis, angiogenesis, and inhibition of apoptosis [63]. Activation of Src can occur through receptor overexpression, and less frequently, through mutation. Elevated levels of activated Src have been well-documented in various cancers, including breast cancer, non-small cell lung cancer, leukemia, colon cancer, gliomas, and other solid tumors [62,[64], [65], [66]]. The study also identified the Sp1 transcription factor as a key regulator that directly interacts with EGFR and SRC proteins. It has been reported that Sp1 binds to the promoter of the Tissue Inhibitor of Matrix Metalloproteinase 1 (TIMP1) and triggers its expression in GBM. TIMP1 is significantly upregulated during carcinogenesis, particularly during inflammation and tissue damage [67].

While these findings provide valuable insights into potential therapeutic targets for GBM, certain limitations should be acknowledged. The reliance on three databases for gene identification may limit the comprehensiveness of the analysis, and the phytochemical screening could be expanded in future research to include a broader range of compounds. Additionally, the results should be further validated through in vitro and in vivo studies to confirm the therapeutic potential of the identified phytochemicals.

## 5 Conclusion

This study employed an integrated approach combining network pharmacology and structural biology to identify candidate ligands and key target genes against GBM. The results indicate significant associations between the hub genes EGFR, PIK3CA, and SRC with GBM-related biological, cellular, and molecular functions. The pharmacodynamic analyses revealed that Isochaihulactone and Vismione B form stable protein-ligand complexes, particularly through interactions with critical residues such as Lys298 and Asp933. The consistency between molecular docking, bioinformatics analysis, and network pharmacology underscores the effectiveness of this multidisciplinary approach. Finally, the molecular computational insights gained from this study suggest that, following future experimental studies, the identified phytochemicals may hold promise as preclinical candidates for GBM management, potentially contributing to the ongoing efforts in drug discovery and development for this challenging cancer.

## CRediT authorship contribution statement

**Hafiza Maria Usmani Rana:** Writing – original draft, Formal analysis, Data curation. **Haseeb Nisar:** Writing – original draft, Supervision, Data curation, Conceptualization. **Jignesh Prajapati:** Software, Resources, Formal analysis, Data curation. **Dweipayan Goswami:** Software, Resources. **Ravi Rawat:** Software, Resources, Investigation. **Volkan Eyupoglu:** Software, Investigation. **Samiah Shahid:** Writing – review & editing, Visualization, Supervision. **Anum Javaid:** Writing – original draft, Software, Formal analysis. **Wardah Nisar:** Writing – original draft, Project administration, Formal analysis, Conceptualization.

## Submission declaration and verification

The work described is not under consideration for publication elsewhere, and its publication is approved by all authors if accepted, it will not be published elsewhere in the same form, in English or any other language, including electronically without the written consent of the copyright-holder.

## Ethics declarations

Not applicable.

## Data availability statement

Digenet: Glioblastoma Multiforme associated genes.

GeneCards: Glioblastoma Multiforme associated genes.

GSE12657: GEO Dataset GSE12657.

GSE86202: GEO Dataset GSE86202.

NPACT Database: NPACT Database for Phytochemicals.

## Funding statement

This research did not receive any specific grant from funding agencies in the public, commercial, or not-for-profit sectors.

## Declaration of competing interest

The authors declare that they have no known competing financial interests or personal relationships that could have appeared to influence the work reported in this paper.

## Figures and Tables

**figs1:**
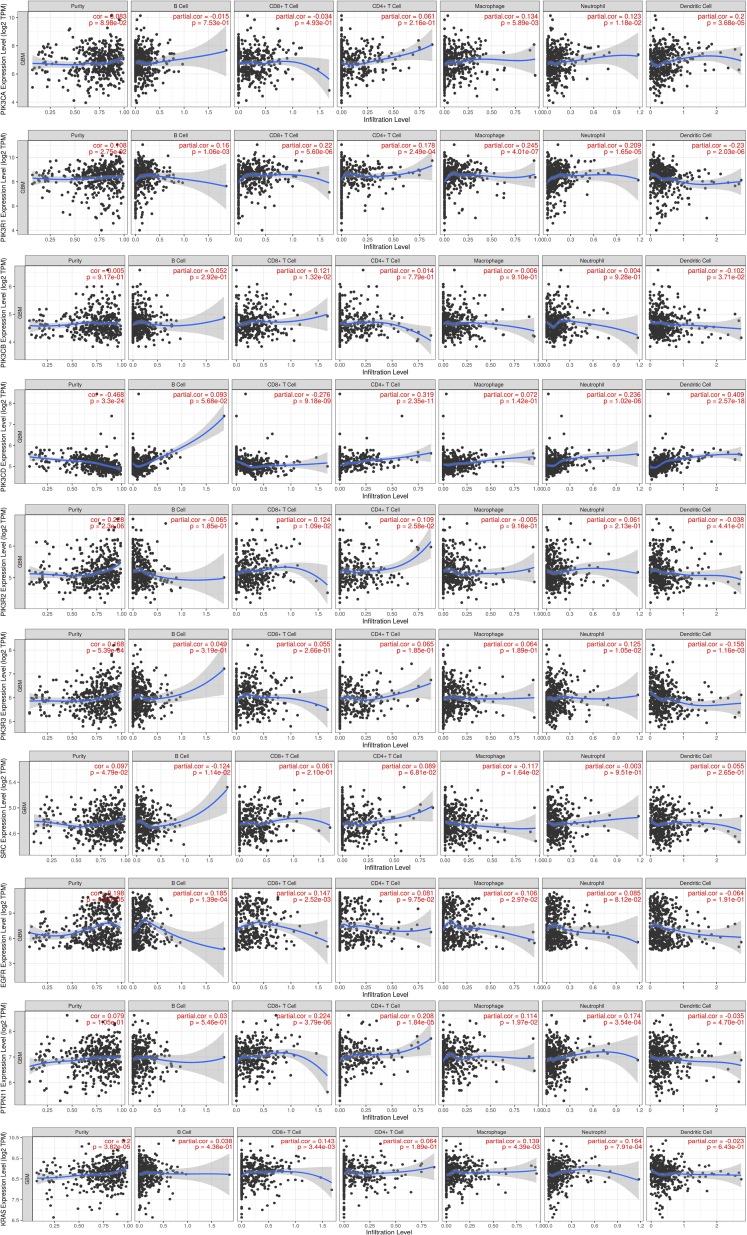


**figs2:**