# Supplementary material for: Integrative bioinformatic analysis to identify potential phytochemical candidates for glioblastoma
Source: Heliyon. 2024 Dec 5;10(24):e40744. doi: 10.1016/j.heliyon.2024.e40744 (PMC11665539; doi:10.1016/j.heliyon.2024.e40744)
Supplement: Multimedia component 3 [file mmc3.docx]

| Chemical Name | Chemical Structure |
| --- | --- |
| "9-alpha-hydroxyparthenolide | 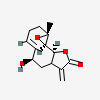 |
| 9-beta-hydroxyparthenolide | 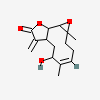 |
| 2-acetylfuro-1,4-naphthoquinone | 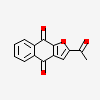 |
| Isochaihulactone | 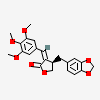 |
| Resveratrol | 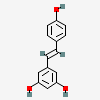 |
| Liriodenine | 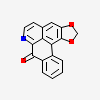 |
| Mitrekaurenone | 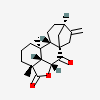 |
| Oropheolide | 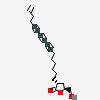 |
| Vismione B | 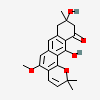 |

a
