# Supplementary material for: Integrative bioinformatic analysis to identify potential phytochemical candidates for glioblastoma
Source: Heliyon. 2024 Dec 5;10(24):e40744. doi: 10.1016/j.heliyon.2024.e40744 (PMC11665539; doi:10.1016/j.heliyon.2024.e40744)
Supplement: Multimedia component 4 [file mmc4.docx]

|  | Liriodenine | 9-alpha-hydroxyparthenolide | 9-beta-hydroxyparthenolide | 2-acetylfuro-1,4-naphthoquinone | Isochaihulactone | Resveratrol | Liriodenine | Mitrekaurenone | Oropheolide | Vismione B |
| --- | --- | --- | --- | --- | --- | --- | --- | --- | --- | --- |
| AMES toxicity | Yes | Yes | Yes | Yes | No | Yes | Yes | No | No | No |
| Max tolerated dose (human) | 0.332 | 0.17 | 0.17 | 0.281 | 0.216 | 0.331 | 0.322 | -0.275 | -0.494 | -0.091 |
| hERG I inhibitor | No | No | No | No | No | No | No | No | No | No |
| hERG II inhibitor | No | No | No | No | No | No | No | No | No | No |
| Oral Rat Acute Toxicity (LD50) | 2.926 | 2.572 | 2.572 | 2.106 | 2.493 | 2.529 | 2.926 | 2.017 | 1.494 | 2.664 |
| Oral Rat Chronic Toxicity (LOAEL) | 1.803 | 2.245 | 2.245 | 1.942 | 1.274 | 1.533 | 1.803 | 1.734 | 2.154 | 1.578 |
| Hepatotoxicity | Yes | No | No | No | No | No | Yes | Yes | No | Yes |
| Skin Sensitisation | No | No | No | No | No | No | No | No | No | No |
| *T.Pyriformis* toxicity | 0.297 | 0.448 | 0.448 | 0.723 | 0.36 | 0.746 | 0.297 | 0.493 | 1.397 | 0.594 |
| Minnow toxicity | 1.038 | 2.346 | 2.346 | 1.091 | -1.429 | 1.522 | 1.038 | 0.246 | -0.416 | 0.718 |

**Supplementary Table 3: Toxicity analysis of selected drugs through pkCSM**
